# Effects of Premortem Stress on Protein Expression, Steak Color, Oxidation, and Myofibrillar Fragmentation Index in the *Longissimus Lumborum*

**DOI:** 10.3390/ani14152170

**Published:** 2024-07-25

**Authors:** Reganne K. Briggs, Jerrad F. Legako, Paul R. Broadway, Jeff A. Carroll, Nicole C. Burdick Sanchez, Nikole E. Ineck, Zachary K. Smith, Ranjith Ramanathan, Kara J. Thornton

**Affiliations:** 1 Animal Dairy and Veterinary Sciences, Utah State University, Logan, UT 84322, USA; reganne.briggs@usu.edu (R.K.B.); nikoleeineck@gmail.com (N.E.I.); 2 Animal and Food Sciences, Texas Tech University, Lubbock, TX 79409, USA; jerrad.legako@ttu.edu; 3 USDA-ARS Livestock Issues Research Unit, Lubbock, TX 79403, USA; rand.broadway@usda.gov (P.R.B.); jeff.carroll@usda.gov (J.A.C.);; 4 Animal Science, South Dakota State University, Brookings, SD 57007, USA; zachary.smith@sdstate.edu; 5 Animal and Food Sciences, Oklahoma State University, Stillwater, OK 74078, USA; ranjith.ramanathan@okstate.edu

**Keywords:** beef, heat shock proteins, oxidation, premortem stress, steak color

## Abstract

**Simple Summary:**

This study aimed to determine the effects of premortem stress on beef quality following harvest. Forty castrated Holstein calves underwent an adrenocorticotropic hormone (ACTH) challenge to emulate a stress response. The calves were harvested at different times (2, 12, 24, and 48 h) following the challenge. In addition, cortisol was measured to determine the specific stress response of each animal during the ACTH challenge. Beef quality attributes such as the breakdown of myofibrillar proteins, color, and pH were analyzed in samples at different ages following harvest. The results show that harvest time following the ACTH challenge does impact the quality of beef that is produced. Additionally, steak color, tenderness, and protein expression may be related to stress that occurs prior to harvest.

**Abstract:**

Forty castrated Holstein calves underwent an adrenocorticotropic hormone (ACTH) challenge to assess the effects of premortem stress on the *longissimus lumborum* (LL) following harvest. LL biopsies were collected before the challenge, at different harvest times (2, 12, 24, and 48 h; *n* = 10), and after 14 d aging. The expression of small heat shock proteins (SHSPs), deglycase 1 (DJ-1), and troponin were analyzed. Blood was analyzed throughout the ACTH challenge and at harvest for cortisol, oxidative stress, and complete blood count (CBC). Color and myofibrillar fragmentation index (MFI) were measured in aged samples. Unexpectedly, calves from different harvest times differed (*p* = 0.05) in cortisol response. Calves were divided into two different cortisol response groups (high or low; *n* = 20). Statistical analysis assessed the effects of cortisol response (*n* = 20), harvest time (*n* = 10), and their interaction. Harvest time altered SHSPs (*p* = 0.03), DJ-1 (*p* = 0.002), and troponin (*p* = 0.02) expression. Harvest time and cortisol response impacted steak color (*p* < 0.05), and harvest time altered steak pH (*p* < 0.0001). Additionally, various CBCs were changed (*p* < 0.05) by harvest time. Harvest time changed (*p* = 0.02) MFI. These data demonstrate that the protein expression, color, and MFI of the LL may be influenced by premortem stress.

## 1. Introduction

Tenderness and color are two of several different quality attributes that influence consumer satisfaction and purchasing decisions [1,2,3]. Despite similar production practices, meat products from beef cattle exhibit undesirable variation in tenderness and stability of flavor and color [4,5]. These inconsistencies may be a result of the effect that external factors, such as premortem stress, elicit on heat shock proteins (HSPs) and oxidative stress [6,7]. 

Heat shock proteins are abundant and highly conserved proteins that help mitigate the effects of various stressors [8]. Some HSPs are constitutively expressed, while others are upregulated in response to stressful conditions to protect proteins from damage [9]. Small heat shock proteins (SHSPs) are upregulated in response to stressful conditions and are ATP-independent, so they may still be active after harvest [7,10,11]. Recent research suggests that SHSPs may play a role in the development of beef tenderness and color; however, their exact role is unknown [6,7,12]. 

Oxidative stress following a stressful event may also play a role in the development of tenderness, color stability, and the flavor of meat from beef cattle. Like tenderness, color is also an important characteristic that consumers consider while purchasing beef [13]. Oxidation causes color deterioration, undesirable flavors, and rancidity development in beef [14,15]. There are many different methods through which oxidative stress can be assessed. Lipid peroxidation forms byproducts known as thiobarbituric acid reactive substances (TBARSs), one of which is malondialdehyde (MDA) [16]. Malondialdehyde is a commonly known marker of oxidative stress [17]. Additionally, there are several different proteins that are known to be involved in oxidative stress. 

The protein deglycase 1 (DJ1) is involved in cellular protection from apoptosis, and several studies have demonstrated that its abundance is related to beef tenderness [18,19,20,21]. Premortem stress may cause a cascading effect of certain pathways to decrease the quality of meat due to decreased tenderness and color stability; however, the effects that premortem stress has on SHSPs and oxidative stress are poorly understood. As such, the goal of this research was to understand how the time of harvest following premortem stress impacts biological pathways involved in stress, proteolysis, and color development in the *longissimus lumborum* (LL). It was hypothesized that as more time elapsed following a stressful event premortem, there would be decreased expression of SHSPs and less oxidative stress as the muscle returned to a homeostatic state, resulting in improved beef quality.

## 2. Materials and Methods 

All experiments with animals were conducted following procedures approved by the USDA-ARS Livestock Issues Research Unit (LIRU) Care and Use Committee under protocol #1808S. A total of forty castrated Holstein calves (103.5 kg ± 1.6), approximately three months old, were obtained from a single commercial source for this study. Although steers at industry harvest weight and of beef breeds would have been ideal for analyzing beef quality parameters, castrated Holstein calves were used based on ease of handling, lower amounts of handling-induced stress, and availability. After arriving at the USDA-ARS LIRU research complex, castrated calves were given one week to acclimate to individual pens, while having ad libitum access to a general grower ration and water. All castrated calves were housed in an indoor, thermoneutral, climate-controlled facility throughout the duration of the study. In addition, animals were not restricted feed regardless of harvest time, and all animals were harvested in the facility where the study took place. 

### 2.1. Initial Skeletal Muscle Samples

After the one-week acclimation period, skeletal muscle biopsies were collected from the right side of the LL at the last rib, following previously described methods [22]. This sample served as an initial control prior to the ACTH injection. In brief, castrated calves were led calmly to a cattle chute and were immobilized. The loin area of each animals was clipped, washed, and disinfected. Lidocaine was given to anesthetize the area before performing a 1 cm incision using a sterile scalpel to expose the muscle. An approximately 2 g sample was collected and was immediately snap-frozen in liquid nitrogen and stored at −80 °C for subsequent analyses. 

### 2.2. ACTH Injection

**Figure 1:**
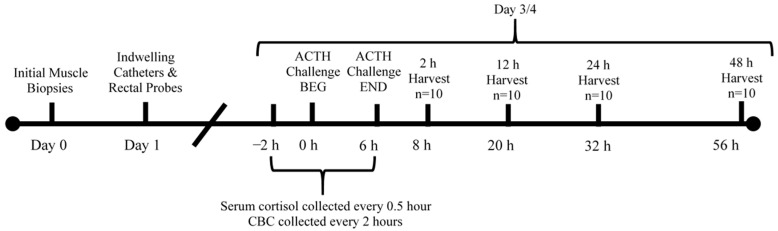
Timeline of sample collection during the adrenocorticotropic hormone (ACTH) challenge. CBC = complete blood count, BEG = beginning of ACTH challenge, END = end of ACTH challenge.

Prior to the ACTH injection, castrated calves were fitted with a rectal temperature monitoring device, as previously described [23], and an indwelling jugular vein catheter, as previously described [24], for serial blood collection. The following day, all calves were injected intravenously with porcine ACTH at a dose of 0.1 IU/kg of body weight (Bachem Chemicals, Torrance, CA, USA) to initiate a stress response. Following the ACTH challenge, castrated calves were serially harvested in groups of 10 animals at the following time points after 6 h exposure to the ACTH-induced stress response: 2 h, 12 h, 24 h, and 48 h (Figure 1). 

### 2.3. Blood Analyses

A ProCyte DX hematology analyzer (IDEXX Laboratories, Westbrook, ME, USA) was used to measure complete blood count (CBC) every 2 h from −2 to 6 h relative to the administration of the ACTH injection at 0 h. In addition, serum was collected for analysis of cortisol every 0.5 h from −2 to 6 h relative to the administration of the ACTH challenge at 0 h; this was stored at −80 °C until analyzed. Serum cortisol concentrations were determined using an enzyme immunoassay (Arbor Assays, Ann Arbor, MI, USA) according to the manufacturer’s instructions. Intra- and inter-assay coefficients of variation were less than 8% and 12%, respectively. Following the collection of blood, all catheters were flushed with 5 mL of saline (0.9% *w*/*v* NaCl) followed by 5 mL of heparinized saline (1 mL of heparin 10,000 IU/mL in 500 mL of saline) to replace fluid volume and maintain catheter patency. 

### 2.4. Sample Collection

At harvest, serum was collected from blood samples. Blood collected at harvest will be subsequently referred to as the “final” sample. Within 30 m of exsanguination, an approximately 5 g skeletal muscle sample from the right side of the LL was collected, approximately 5 cm anterior from the initial biopsy site. All muscle samples collected for protein and oxidative measures were immediately snap-frozen in liquid nitrogen and were stored at −80 °C for subsequent analysis. The whole LL from the left side was excised and vacuum packaged, and was then held at refrigeration (2–4 °C) for 14 d until a final LL muscle sample was collected from the posterior portion of the muscle.

### 2.5. Western Blotting

Skeletal muscle samples were ground under liquid nitrogen, and total protein was extracted following previously described procedures [25]. In brief, ground tissue was added to total protein extraction buffer (50 mM Tris-HCl (pH 7.52), 150 mM NaCl, 1 mM ethylenediaminetetraacetic acid (EDTA), 1% Tergitol, 0.1% SDS, and 0.5% sodium deoxycholate) containing phosphatase and protease inhibitor tablets (Roche, Indianapolis, IN, USA). Samples were homogenized and rocked for 10 min at 4 °C. Samples were centrifuged at 10,000× *g* for 10 min at 4 °C. The supernatant was removed and stored at −80 °C. Total protein was quantified using a Pierce™ BCA Protein Assay Kit (Thermo Scientific, Waltham, MA, USA). Proteins (0.3 µg total protein added per lane) were separated using SDS—10% polyacrylamide gels for 90 min at 140 V at 4 °C. Proteins were transferred to an immobilidon-polyvinylidene fluoride (PVDF) membrane at 4 °C for 90 min at 100 V. Western blot analyses were completed on samples collected prior to the ACTH challenge, immediately following harvest, and after 14 d of aging to measure the abundance of HSPβ1, phosphorylated-HSPβ1 (P-HSPβ1), HSPβ5, DJ1, and troponin I using specific primary and secondary antibodies (Table 1). 

**Figure 2:**
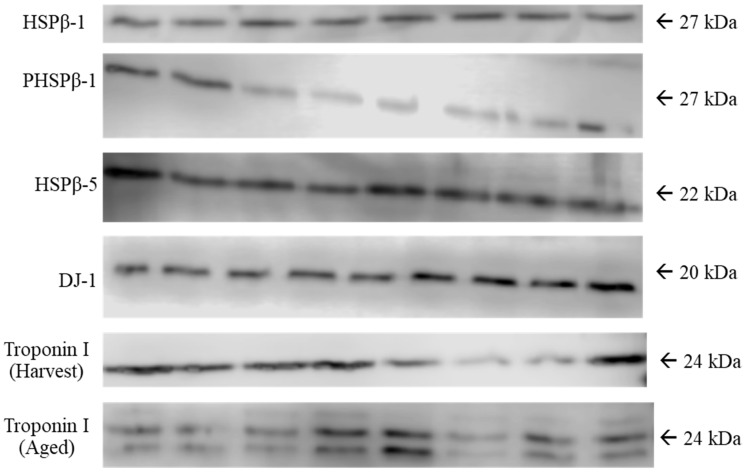
Representative Western blot images from the castrated Holstein calves. The representative proteins are heat shock protein β-1 (HSPβ-1), phosphorylated heat shock protein β-1 (PHSP-β1), protein deglycase (DJ-1), and troponin at harvest and after 14 d of aging. Samples were randomized by treatment groups and harvest time across each blot. Each blot is a representation of each target protein that was analyzed.

All membranes were blocked in 5% non-fat milk plus 1X tris-buffered saline (1X TBS) for 1 h at room temperature, and were then incubated in primary antibody overnight at 4° C. Membranes were washed in 1X TBS and were incubated with secondary anti-rabbit antibody (HSPβ1, P-HSPβ1, DJ1, and troponin I) (7074S, Cell Signaling Technology, Beverly, MA, USA) or anti-mouse antibody (HSPβ5) (31430, Invitrogen, Rockford, IL, USA) conjugated to horseradish peroxidase (HRP) for 2 h at room temperature. Bands were visualized using a C-DiGit^®^ Blot Scanner (LI-COR, Lincoln, NE, USA) and were quantified with Image Studio™ (LI-COR, Lincoln, NE, USA). One skeletal muscle sample not associated with this study was run on each blot; this was considered as the internal standard to account for differences between different blots. Protein abundance values were adjusted to the internal standard. See Figure 2 for the representative blots and target proteins analyzed. Blots were randomized by treatment and harvest time. As such, samples in different treatment groups were not compared visually across the same blot.

### 2.6. Myofibrillar Fragmentation Index

Skeletal muscle from samples aged for 14 d postmortem were ground under liquid nitrogen, and myofibril fragments were extracted as previously described [26]. In brief, ground tissue was added to myofibrillar fragmentation index (MFI) buffer (100 mM KCl, 20 mM KPO4-pH 7, mM EGTA, 1 mM MgCl2, and 1 mM NaN3) and was homogenized for 30 s. Samples were centrifuged twice at 1000× *g* for 15 min at 4 °C; supernatant was decanted, and fresh MFI buffer was added each time. Myofibrillar fragments were then quantified using the Pierce™ BCA Protein Assay Kit (Thermo Scientific, Waltham, MA, USA). After quantification, samples were diluted, plated, and read at 540 nm on a BioTek Synergy H1 plate reader (Agilent, Santa Clara, CA, USA) to determine absorbance. All assays were run in triplicate and the average value was reported. The measurement of MFI = 200 × absorbance.

### 2.7. Color and pH

Steaks aged for 14 d were used for retail display color analysis. Steak color was measured on d 0, 1, 2, 3, and 4. Steaks were placed onto foam trays with absorbent pads and were over-wrapped with a PVC film (oxygen-permeable polyvinyl chloride fresh meat film; 15,500 to 16,275 cm^3^ O_2_ m^−2^ 24 h^−1^ at 23 °C, E-Z Wrap Crystal Clear Polyvinyl Chloride Wrapping Film, Koch Supplies, Kansas City, MO, USA). After packaging, steaks were placed in a coffin-style open display case maintained at 2 °C ± 1 under continuous lighting (1612 to 2152 lx, Philips Deluxe Warm White Fluorescent lamps; Andover, MA, USA; color rendering index = 86; color temperature = 3000 K). All packages were rotated daily to minimize variances in light intensity and/or temperature caused by specific case locations. The surface color was measured using a HunterLab MiniScan XE Plus spectrophotometer (Model 45/0 large area view, 2.5 cm diameter aperture, Illuminant A, 10° Observer; HunterLab, Reston, VA, USA) on respective display days. Both reflectance spectra from 400 to 700 nm (10 nm increments) and CIE L*, a*, and b* values were measured on each steak at three random locations, and subsamples were averaged for statistical analyses. The pH of each steak was recorded using an Accumet 50 pH meter (Fisher Scientific, Fairlawn, NJ, USA) in duplicate, and the values were averaged.

### 2.8. TBARSs

TBARSs were measured in serum at 2 h increments throughout the study and at exsanguination utilizing a modification of previously described methods [27,28]. A standard curve was prepared using a trichloroacetic acid (TCA)/thiobarbituric acid (TBA) reagent (15% TCA (*w*/*v*) and 20 mM TBA in DI water) and a diluted 1, 1, 3, 3-tetra-ethoxypropanone (TEP) standard. Samples were ground under liquid nitrogen, and 2.5 g was added to 7.5 mL of cold DI water. Samples were then vortexed for 90 s and were then centrifuged for 15 min at 4500× *g*. Following centrifugation, 2 mL of supernatant was added to 4 mL of TCA/TBA reagent; then, 100 µL of butylated hydroxyanisole (BHA) was added. Samples were then vortexed for approximately 1 min. For the serum, 250 µL of sample was added to 500 µL of TCA/TBA reagent and 10 µL of BHA and was vortexed for approximately 1 min. All prepared samples and standards were heated for 1 h at 90 °C, and were then submerged in an ice water bath for 20 min. For standards, tubes were then brought to ambient temperature before each standard was plated for evaluation. Heated and cooled serum and tissue samples were centrifuged for 15 min at 4500× *g*, then 200 µL of supernatant was plated onto a 96-well plate for evaluation at 531 nm. Standard curves were used to determine the concentrations of MDA in all samples.

### 2.9. Statistical Analysis

**Figure 3:**
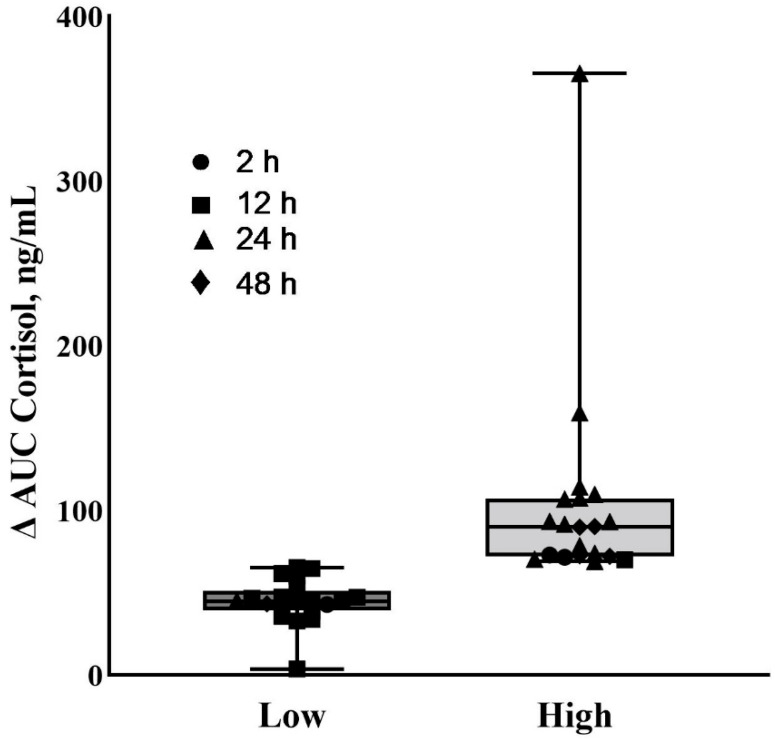
Different quartile groupings of castrated Holstein calves (103.5 kg ± 1.6) based on the delta area under the curve (AUC) cortisol. This was measured by finding the difference between the AUC of cortisol −2 to 0 h before initiation of the adrenocorticotropic hormone (ACTH) challenge (0.1 IU/kg of body weight) to initiate a stress response and the AUC of cortisol 0 to 2 h after ACTH was given. Castrated calves were grouped as having a low or high delta AUC cortisol (*n* = 20). Differently shaped data points represent different harvest times following ACTH challenge. Individual data points represent the delta AUC cortisol response of each individual castrated calf within each group. Shaded boxes represent the delta AUC cortisol. Error bars represent the minimum and maximum data point in each cortisol response group.

Statistical analysis for all measurements was performed using the MIXED Procedure of SAS version 9.4 (SAS Institute Inc., Cary, NC, USA). The main effect of time of harvest (*n* = 10) was analyzed with each individual calf as a random variable. The differences in cortisol response after the initiation of the ACTH challenge was not anticipated by the authors. It was hypothesized that cortisol response would be similar amongst groups, as they were randomly placed into harvest groupings and had similar backgrounds. However, since the cortisol response was found to differ (*p* < 0.05) among harvest groups 0.5 h after administration of ACTH, the effect of the ACTH-induced cortisol response was included as a fixed effect in the model. A total of two cortisol response groups (high or low), with 20 calves per group (*n* = 20), were assigned based on the delta area under the curve (AUC) for cortisol. This was calculated by measuring the difference in the AUC for serum cortisol concentration collected every 0.5 h from −2 to 0 h and 0 to 2 h relative to ACTH challenge (Figure 3). The effect of the delta AUC of cortisol will be referred to as cortisol response throughout the paper. The final statistical model assessed the main effects of harvest time, cortisol response, and their interaction. Protein abundance was found to be different in samples collected before the ACTH challenge, as such relative abundance was calculated and used in statistics to account for individual animal variation. Relative protein abundance was calculated by dividing the initial protein abundance of the skeletal muscle biopsy by the abundance in the samples collected at harvest and after 14 d of aging. Steak color, TBARSs, and CBC were analyzed using repeated measures and PROC MIXED, where harvest time, cortisol response, and their interaction were used as fixed effects and individual calves served as random effects in the model. In all analyses, when treatment differences were found to be significant (*p* < 0.05), least square means were separated using Tukey–Kramer adjustments. A *p* < 0.05 was considered statistically significant, whereas a *p* < 0.10 was considered a trend. All data are presented as the least square mean ± SEM.

## 3. Results

### 3.1. Cortisol 

**Figure 4:**
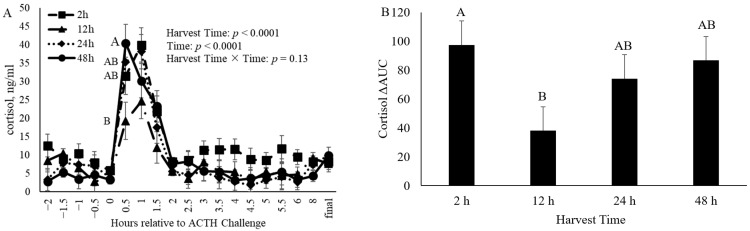
(**A**): Cortisol concentration in serum of castrated Holstein calves (103.5 ± 1.6 kg) relative to the beginning of the adrenocorticotropic hormone (ACTH) challenge given at a dose of 0.1 IU/kg of body weight. Treatment groups consisted of four groups of calves that were harvested at different time points (2 h, 12 h, 24 h, and 48 h; *n* = 10) following the ACTH challenge. Values represent the least squares means ± SEM of cortisol concentration in serum collected relative to the ACTH challenge for harvest times. Points with different letters differ (*p* < 0.05) among harvest times at that specific time point. (**B**): Differences in the delta area under the curve (ΔAUC) of cortisol calculated as the AUC from 0 to 2 h of the challenge–AUC from −2 to 0 h of the challenge between animals harvested at different time points. Bars with different letters indicate a difference (*p* < 0.05) among the different harvest times.

Unexpectedly, the concentration of cortisol in the serum collected every 0.5 h relative to the ACTH challenge was shown to be impacted by both time of harvest (*p* < 0.001) and time (*p* < 0.001) relative to the ACTH challenge (Figure 4). Differences among groups that were to be harvested at different times were present 0.5 h after the initiation of the ACTH challenge, such that calves that were to be harvested 48 h after the ACTH challenge had an increased (*p* < 0.001) serum cortisol concentration compared to calves that were to be harvested 12 h after the ACTH challenge (Figure 4A). However, no differences (*p* > 0.05) among harvest time groups were observed through the remainder of the ACTH challenge. In addition, the cortisol response measured as ΔAUC was different (*p* < 0.05) between the different harvest times, such that animals harvested at 12 h had a decreased (*p* < 0.05) ΔAUC compared to calves harvested at 2 h, but were no different (*p* > 0.05) than those harvested at 24 or 48 h (Figure 4B).

### 3.2. Rectal Temperature

**Figure 5:**
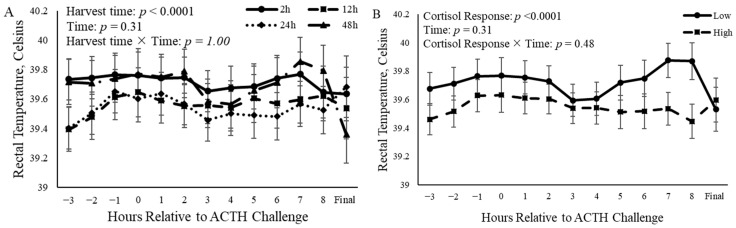
Rectal temperature of castrated Holstein calves (103.5 ± 1.6 kg) relative to the beginning of the ACTH challenge given at a dose of 0.1 IU/kg of body weight. (**A**): Treatment groups consisted of four groups of calves that were harvested at different time points (2 h, 12 h, 24 h, and 48 h; *n* = 10) following the ACTH challenge. (**B**): Calves were grouped based on cortisol response relative to ACTH challenge and were split into two different groups (low or high; *n* = 20). Repeated measures analyses were completed to determine the effects of harvest time, cortisol response, time, harvest time × time, and harvest time × cortisol response. Values represent the least squares mean ± SEM for (**A**) harvest times or (**B**) cortisol responses.

The time of harvest altered (*p* < 0.0001) rectal temperature over time relative to the ACTH challenge (Figure 5A). Castrated calves that were to be harvested 12 h after the ACTH challenge had a decreased (*p* < 0.0001) rectal temperatures compared to calves that were to be harvested 2 h and 48 h after the ACTH challenge (Figure 5A). There was also a relationship (*p* < 0.0001) between cortisol response and rectal temperature, such that calves that had a low cortisol response had an increased rectal temperature compared to calves that had a high cortisol response (Figure 5B).

### 3.3. Protein Abundance 

Quantifications of five proteins using antibody-specific Western blotting (Figure 2) demonstrated that both harvest time and cortisol response affected protein expression. There was no effect (*p* = 0.22) of time of harvest on relative HSPβ1 abundance in samples collected at harvest (Table 2). However, samples aged for 14 d had altered (*p* = 0.03) HSPβ1 abundance in the LL between harvest times (Table 2). In addition, cortisol response did not alter the expression of HSPβ1 at harvest (*p* = 0.29) or after 14 d of aging (*p* = 0.19) (Table 3). 

Time of harvest affected (*p* = 0.03) relative P-HSPβ1 abundance in the LL at harvest, such that castrated calves that were harvested 48 h after the ACTH challenge had an increased (*p* = 0.03) abundance of P-HSPβ1 compared to calves that were harvested 12 h after the ACTH challenge (Table 2). The relative P-HSPβ1 abundance of samples aged for 14 d differed (*p* = 0.001) between harvest times, where calves that were harvested 2 h and 48 h after the ACTH challenge had a decreased (*p* = 0.001) abundance of P-HSPβ1 compared to calves that were harvested 12 h after the ACTH challenge (Table 2). Cortisol response did not impact the expression of P-HSPβ1 at harvest (*p* = 0.15) nor after 14 d of aging (*p* = 0.81) (Table 3). 

Analysis of the expression of HSPβ5 from samples collected at harvest and after 14 d of aging was not different (*p* = 0.67 and *p* = 0.43, respectively) relative to the time of harvest (Table 2). Similarly, analysis of the relative HSPβ5 expression was not altered by cortisol response at harvest (*p* = 0.40) or after 14 d of aging (*p* = 0.25) (Table 3). 

The relative expression of DJ1 was different at both harvest (*p* = 0.002) and after 14 d of aging (*p* < 0.0001) among castrated calves harvested at different times after the ACTH challenge (Table 2). At harvest, calves that were harvested 12 h after the ACTH challenge had a decreased (*p* = 0.002) DJ-1 expression compared to calves that were harvested 24 h after the ACTH challenge. After 14 d of aging, samples from calves that were harvested 24 h after the ACTH challenge had an increased (*p* < 0.0001) expression of DJ1 compared to the other three harvest time groups (Table 2). Cortisol response tended to affect the relative DJ1 expression at harvest and after 14 d of aging in the LL (*p* = 0.07 and *p* = 0.08, respectively) (Table 3).

Time of harvest did not have an effect (*p* = 0.22) on the relative troponin expression in the LL at harvest (Table 2). Further, cortisol response had no effect (*p* = 0.49) on troponin expression at harvest (Table 3). The expression of troponin analyzed after 14 d aging was quantified as the entire band, and the upper and lower bands were quantified as degradation of this protein had occurred during aging. The time of harvest impacted (*p* = 0.01) the expression of the entire band of troponin. Calves that were harvested 48 h after the ACTH challenge had an increased (*p* = 0.01) troponin expression compared to calves that were harvested 12 h after the ACTH challenge (Table 2). The relative expression of the entire band of troponin after 14 d of aging was not impacted (*p* = 0.52) by cortisol response (Table 3). 

The time of harvest affected (*p* = 0.02) the expression of the lower band of troponin I after 14 d of aging, such that samples from calves that were harvested 48 h after the ACTH challenge had an increased (*p* = 0.02) expression compared to samples from calves harvested at 2 h and 12 h after the ACTH challenge (Table 2). Cortisol response did not impact (*p* = 0.88) the lower band of troponin I after 14 d of aging (Table 3). 

### 3.4. Complete Blood Counts 

**Figure 6:**
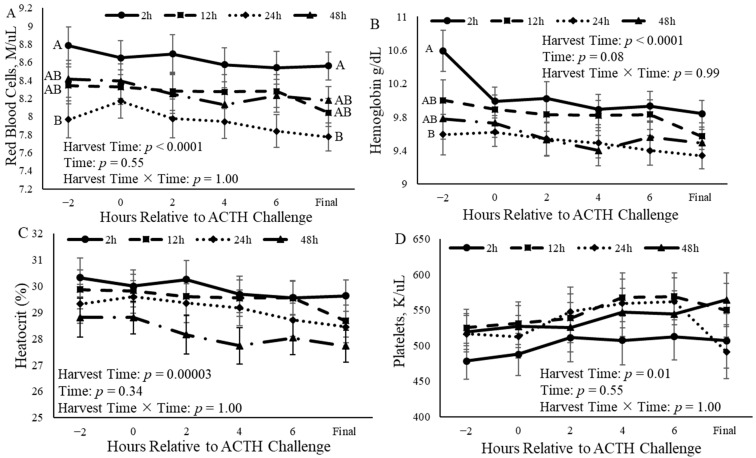
Concentration of blood components of castrated Holstein calves (103.5 ± 1.6 kg) ((**A**): red blood cells; (**B**): hemoglobin; (**C**): hematocrit; (**D**): platelets) measured every 2 h relative to adrenocorticotropic hormone (ACTH) challenge given at a dose of 0.1 IU/kg of body weight between calves grouped into harvest time (2 h, 12 h, 24 h, and 48 h; *n* = 10). Repeated measures analyses were completed to determine the effects of harvest time, time, and harvest time × time. Values represent the least squares mean ± SEM for the respected blood component. Points with different letters differ (*p* < 0.05) among harvest times at each time point.

The time of harvest affected (*p* < 0.0001) the red blood cell (RBC) concentration (Figure 6A). Castrated calves that were to be harvested 24 h after the ACTH challenge had a decreased (*p* < 0.05) concentration compared to calves that were to be harvested at the other three time points (Figure 6A). Additionally, the time of harvest altered (*p* < 0.0001) the hemoglobin concentration, where calves that were to be harvested 2 h after the ACTH challenge had an increased (*p* < 0.05) hemoglobin concentration compared to calves that were to be harvested 24 and 48 h after the ACTH challenge (Figure 6B). Additionally, calves that were to be harvested 12 h after the ACTH challenge had increased (*p* < 0.05) hemoglobin concentrations compared to those that were harvested 24 h after the ACTH challenge (Figure 6B). Furthermore, hematocrit was impacted (*p* = 0.0003) by the time of harvest, such that calves harvested 48 h after the ACTH challenge had a decreased (*p* < 0.05) hematocrit compared to those harvested 2 and 12 h after the challenge (Figure 6C). The platelet concentration was also altered (*p* = 0.01) by the time of harvest, although no differences (*p* > 0.05) among groups that were to be harvested at different times were observed (Figure 6D). The final concentrations of hemoglobin, hematocrit, and platelets collected at harvest did not differ (*p* = 0.10, *p* = 0.18, and *p* = 0.59, respectively) among harvest times; however, RBC concentrations were different (*p* = 0.01). 

**Figure 7:**
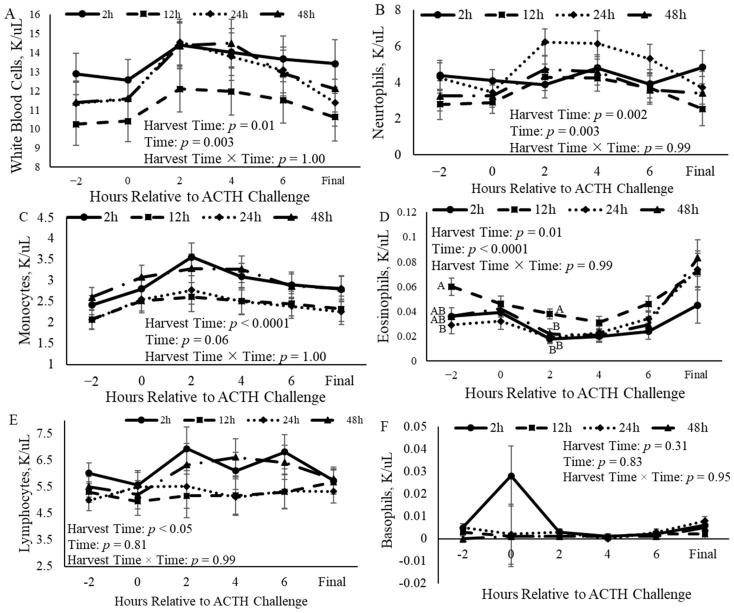
Concentration of blood components of castrated Holstein calves (103.5 ± 1.6 kg) ((**A**): white blood cells, (**B**): neutrophils, (**C**): monocytes, (**D**): eosinophils, (**E**): lymphocytes, (**F**): basophils) measured every 2 h relative to adrenocorticotropic hormone (ACTH) challenge given at a dose of 0.1 IU/kg of body weight between calves grouped into harvest time (2 h, 12 h, 24 h, and 48 h; *n* = 10). Repeated measures analyses were completed to determine the effects of harvest time (treatment), time, and treatment × time. Values represent the least squares mean ± SEM for the respected blood component. Points with different letters differ (*p* < 0.05) among harvest times at each time point.

Concentrations of white blood cells (WBCs), neutrophils, monocytes, eosinophils, and lymphocytes each varied (*p* = 0.01, *p* = 0.002, *p* < 0.0001, *p* = 0.01, and *p* = 0.05, respectively) with the time of harvest (Figure 7). White blood cells were increased (*p* = 0.01) in castrated calves harvested 2 h after the ACTH challenge when compared to calves harvested 12 h after the ACTH challenge. Calves that were harvested at 24 h after the ACTH challenge had increased (*p* = 0.01) neutrophil concentrations compared to calves harvested 12 h after the challenge (Figure 7A). Calves that were harvested 2 h and 48 h after receiving ACTH had increased (*p* = 0.001) monocytes compared to those calves that were harvested 12 h and 24 h after the challenge (Figure 7C). Eosinophils were increased (*p* < 0.0001) in calves harvested 12 h after the ACTH challenge when compared to calves harvested at the three other time points (Figure 7D). Additionally, the concentrations of WBCs, neutrophils, monocytes, and eosinophils changed (*p* < 0.05) over time (Figure 7). In each of these, the concentration increased (*p* < 0.05) 2 h after the ACTH challenge and then decreased after that time point. There was no relationship (*p* = 0.31) between the time of harvest and the concentration of basophils (Figure 7F). The final concentration of WBCs, neutrophils, monocytes, eosinophils, and lymphocytes collected at harvest did not differ (*p* = 0.22, *p* = 0.13, *p* = 0.21, *p* = 0.22, and *p* = 0.93, respectively) among harvest times (Figure 7). However, the final concentration of basophils differed (*p* = 0.006) among harvest times (Figure 7F). 

**Figure 8:**
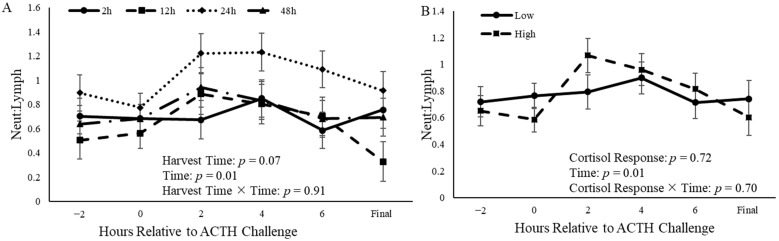
Neutrophil and lymphocyte ratio (neut:lymph) of castrated Holstein calves (103.5 ± 1.6 kg) relative to the beginning of the adrenocorticotropic hormone (ACTH) challenge. (**A**): Calves were grouped based on time of harvest following ACTH challenge given at a dose of 0.1 IU/kg of body weight (2 h, 12 h, 24 h, and 48 h; *n* = 10). (**B**): Calves were grouped based on cortisol response relative to the ACTH challenge and were split into two different groups (low and high; *n* = 20). Repeated measures analyses were completed to determine the effects of harvest time, cortisol response, time, harvest time x time, cortisol response x time, and harvest time × cortisol response. Values represent the least squares mean ± SEM of neut:lymph.

The time of harvest tended to affect (*p* = 0.07) the neutrophil–lymphocyte ratio (N:L) (Figure 8A). However, cortisol response did not affect (*p* = 0.72) the N:L ratio (Figure 8B).

### 3.5. Steak Color 

**Figure 9:**
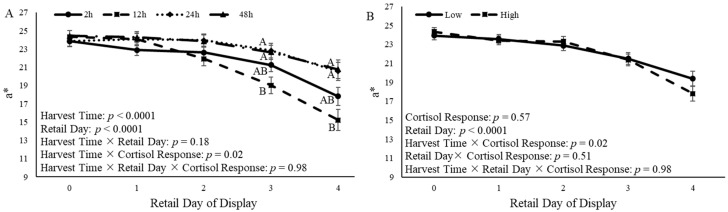
a* (redness) color measurement from the *longissimus lumborum* of castrated Holstein calves (103.5 ± 1.6 kg) after 14 d of aging. (**A**): Calves were grouped based on time of harvest following adrenocorticotropic hormone (ACTH) challenge given at a dose of 0.1 IU/kg of body weight (2 h, 12 h, 24 h, and 48 h; *n* = 10). (**B**): Calves were grouped based on cortisol response relative to the ACTH challenge and were split into two different groups (low and high; *n* = 20). Repeated measures analyses were completed to determine the effects of harvest time, cortisol response, retail day, harvest time × retail day, harvest time × cortisol response, and harvest time × retail day × cortisol response. Values represent the least squares mean ± SEM of a* measurements. Points with different letters differ (*p* < 0.05) among harvest times or cortisol responses at each time point.

A harvest time × cortisol response (*p* = 0.02) was observed for a* values (Figure 9). Additionally, the a* value was affected by the time of harvest (*p* < 0.0001) and retail day (*p* < 0.0001) (Figure 9A). On d 3 and d 4 of retail display, steaks from castrated calves that were harvested 24 h and 48 h after the ACTH challenge had an increased (*p* = 0.03) a* value compared to steaks from calves harvested 12 h after the ACTH challenge (Figure 9A). Cortisol response did not affect (*p* = 0.57) a* values (Figure 9B). 

**Figure 10:**
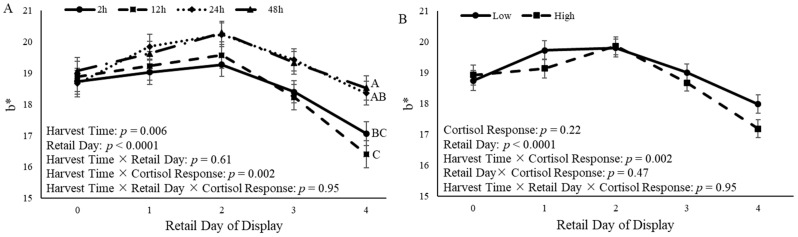
b* (yellowness) color measurement from the *longissimus lumborum* of castrated Holstein calves (103.5 ± 1.6 kg) after 14 d of aging. (**A**): Calves were grouped based on time of harvest following adrenocorticotropic hormone (ACTH) challenge given at a dose of 0.1 IU/kg of body weight (2 h, 12 h, 24 h, and 48 h; *n* = 10). (**B**): Calves were grouped based on cortisol response relative to the ACTH challenge and were split into two different groups (low and high; *n* = 20). Repeated measures analyses were completed to determine the effects of harvest time, cortisol response, retail day, harvest time × retail day, harvest time × cortisol response, and harvest × retail day × cortisol response. Values represent the least squares mean ± SEM of b* measurements. Points with different letters differ (*p* < 0.05) among harvest times or cortisol responses at each time point.

A harvest time × cortisol response (*p* = 0.002) was observed for b* values (Figure 10). The time of harvest (*p* = 0.006) and retail day of display (*p* < 0.0001) each also affected b* values (Figure 10A). After 4 d of retail display, steaks from castrated calves that were harvested 24 h and 48 h after the ACTH challenge had increased (*p* = 0.006) b* values compared to steaks from calves harvested 12 h after the ACTH challenge (Figure 10A). Cortisol response did not affect (*p* = 0.22) b* values (Figure 10B).

**Figure 11:**
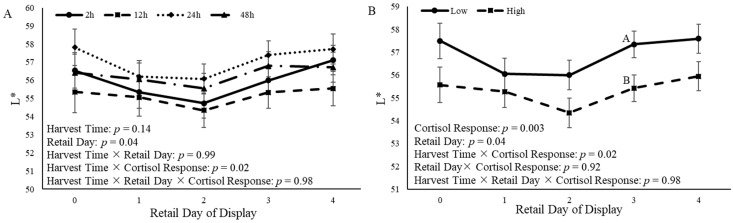
L* (lightness) color measurement from the *longissimus lumborum* of castrated Holstein calves (103.5 ± 1.6 kg) after 14 d of aging. (**A**): Calves were grouped based on time of harvest following adrenocorticotropic hormone (ACTH) challenge given at a dose of 0.1 IU/kg of body weight (2 h, 12 h, 24 h, and 48 h; *n* = 10). (**B**): Calves were grouped based on cortisol response relative to the ACTH challenge and were split into two different groups (low and high; *n* = 20). Repeated measures analyses were completed to determine the effects of harvest time, cortisol response, retail day, harvest time × retail day, harvest time × cortisol response, and harvest × retail day × cortisol response. Values represent the least squares mean ± SEM of L* measurements. Points with different letters differ (*p* < 0.05) among harvest times or cortisol responses at each time point.

Additionally, L* values were impacted by harvest time × cortisol (*p* = 0.02). However, the values for L* were not affected (*p* = 0.14) by the retail display time relative to harvest time (Figure 11A). Additionally, the cortisol response altered (*p* = 0.003) L* values over time. Castrated calves with a low cortisol response had increased (*p* = 0.05) L* values compared to calves with a high cortisol response on d 3 of retail display (Figure 11B).

### 3.6. Steak pH

**Figure 12:**
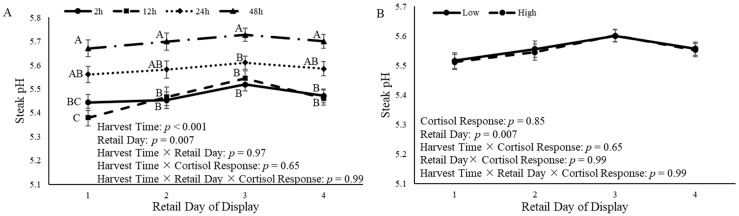
Steak pH measurement from the *longissimus lumborum* of castrated Holstein calves (103.5 ± 1.6 kg) after 14 d of aging. (**A**): Calves were grouped based on time of harvest following adrenocorticotropic hormone (ACTH) challenge given at a dose of 0.1 IU/kg of body weight (2 h, 12 h, 24 h, and 48 h; *n* = 10). (**B**): Calves were grouped based on cortisol response relative to the ACTH challenge and were split into two different groups (low and high; *n* = 20). Repeated measures analyses were completed to determine the effects of harvest time, cortisol response, retail day, harvest time × retail day, harvest time × cortisol response, and harvest × retail day × cortisol response. Values represent the least squares mean ± SEM of steak pH measurements. Points with different letters differ (*p* < 0.05) among harvest times or cortisol responses at each time point.

The steak pH was affected by both harvest time and retail day of display (*p* < 0.0001 and *p* = 0.007, respectively) (Figure 12A). Steaks from castrated calves that were harvested 48 h after the ACTH challenge had greater (*p* < 0.0001) pH values than steaks from calves that were harvested 2 h and 12 h after the ACTH challenge at 1 d and 2 d of retail display (Figure 12A). At 3 d and 4 d of retail display, steaks from calves that were harvested 48 h after the ACTH challenge had greater (*p* < 0.0001) pH values compared to steaks from calves that were harvested 2 h, 12 h, and 24 h after the ACTH challenge (Figure 12A). However, cortisol response did not (*p* = 0.85) impact steak pH (Figure 12B).

### 3.7. Oxidation

**Figure 13:**
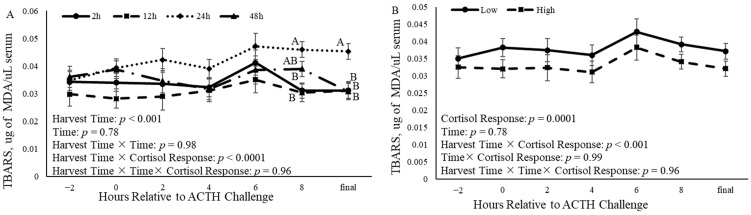
Concentration of TBARSs in serum of castrated Holstein calves (103.5 ± 1.6 kg) collected every 2 h relative to adrenocorticotropic hormone (ACTH) challenge given at a dose of 0.1 IU/kg of body weight. Panel (**A**) describes TBARSs concentration in serum relative to the ACTH challenge in calves that were harvested at different time points (2 h,12 h, 24 h, and 48 h; *n* = 10) following the ACTH challenge. Panel (**B**) describes TBARSs concentration in serum relative to the ACTH challenge in calves that had different cortisol responses (low and high; *n* = 20) relative to the ACTH challenge. Repeated measures analyses were completed to determine the effects of harvest time, cortisol response, time, harvest × time, time x cortisol response, harvest time × cortisol response, and harvest time × time × cortisol response. Values represent the least squares mean ± SEM of TBARSs concentration in the serum. Points with different letters differ (*p* < 0.05) within harvest time or cortisol response at each time point.

The harvest time × cortisol response altered (*p* < 0.0001) serum TBARSs (Figure 13). Additionally, both harvest time (*p* < 0.0001) and cortisol response (*p* < 0.0001) affected serum concentrations of TBARSs (Figure 13). At 8 h relative to the ACTH challenge, concentrations of TBARSs were increased (*p* = 0.001) in the serum of castrated calves harvested 24 h after the ACTH challenge when compared to those harvested 2 and 12 h after (Figure 13A). Final serum TBARSs increased (*p* = 0.0009) in calves harvested 24 h after the ACTH challenge compared to the other three harvest times (Figure 13A). Additionally, cortisol response had an effect (*p* = 0.0001) on TBARSs concentration, such that calves that had a low concentration of cortisol had increased concentrations of TBARSs compared to calves that had a high cortisol response (Figure 13B). 

### 3.8. Myofibrillar Fragmentation Index

**Figure 14:**
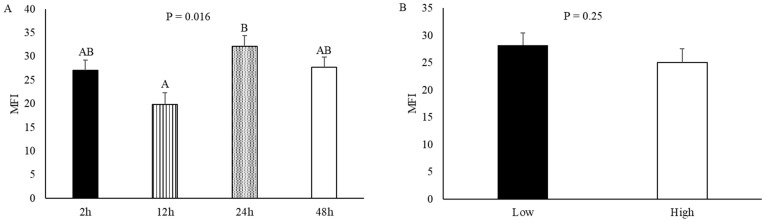
Myofibrillar fragmentation index (MFI) of 14 d aged muscle tissue collected from the *longissimus lumborum* of castrated Holstein calves (103.5 ± 1.6 kg). Panel (**A**) describes the MFI of samples collected from calves that were harvested at different time points (2 h, 12 h, 24 h, and 48 h; *n* = 10) following adrenocorticotropic hormone (ACTH) challenge given at a dose of 0.1 IU/kg of body weight. Panel (**B**) describes the MFI of samples collected from calves that had different cortisol responses (low and high; *n* = 20) after the initiation of the ACTH challenge. Values represent the least squares mean ± SEM of MFI for 14 d aged muscle tissue. The *p*-value above each time point represents the effect of harvest time (**A**) or cortisol response (**B**). Bars with different letters are different (*p* < 0.05) from one another.

The time of harvest changed (*p* = 0.016) MFI, such that castrated calves that were harvested 24 h after the ACTH challenge had an increased (*p* < 0.05) MFI value compared to calves that were harvested 12 h after the ACTH challenge (Figure 14A). However, cortisol response did not affect (*p* = 0.25) MFI (Figure 14B).

## 4. Discussion

The rate and extent of the postmortem breakdown of myofibrillar proteins are major determinants of tenderness [20,29]. Although the development of end-product tenderness is a well-researched area, inconsistencies in tenderness still exist [30]. Premortem stress may be involved in decreased meat quality by modulating HSP abundance. Heat shock proteins are highly conserved proteins that can be constitutively expressed or upregulated during stressful conditions [8,9], and act as molecular chaperones to maintain cellular homeostasis [8]. Small heat shock proteins play a similar role, acting as molecular chaperones, but are ATP-independent, indicating that they can still be active postmortem [31,32]. Proteomic studies of muscle have identified that some SHSPs are upregulated in postmortem muscle [33]. Following a stressful event, oxidative stress may affect the tenderness, color stability, and flavor of meat by causing color deterioration, undesirable flavors, and rancidity in meat [15]. As such, the goal of this research was to understand how biological pathways, proteolysis, and color are changed in beef following a stressful event prior to harvest. To study the effects of acute stress, such as transport, castrated calves were challenged with ACTH prior to being serially harvested. Differences in cortisol concentrations 0.5 h after initiation of the ACTH challenge were observed between harvest groups; therefore, cortisol response was also included as a main effect in the model. It should be mentioned that the difference in serum cortisol concentration was not anticipated by the authors.

The calves used in the present study were much smaller and younger than industry-standard finished feedlot cattle. In addition, the present study used a dairy breed rather than a beef breed. To the authors’ knowledge, no previous research has analyzed beef quality traits on castrated calves of this size or age in relation to stress. As such, many comparisons are made with beef breeds and/or finished feedlot cattle throughout the discussion.

In the present study, most blood components in the serum differed over time based on the time of harvest relative to the ACTH challenge. Calves allowed 2 h of rest following the ACTH challenge had an increased concentration of WBCs compared to calves harvested 12 h after the challenge. However, the concentration of WBCs from calves that were harvested 48 h after the challenge did not differ from other harvest times. However, a study involving Limousine feedlot beef cattle that were transported for 5 h observed an increased WBCs concentration 48 h following transportation [34]. In the present study, the platelet count was not affected by harvest time; however, hematocrit was increased in calves that were harvested 2 h and 12 h after the ACTH challenge compared to calves that were harvested 48 h after the ACTH challenge. This could be a result of minor dehydration due to stress during the ACTH challenge, although calves had ad libitum access to water until harvest. The current study showed a slight increase in eosinophil concentration at harvest compared to eosinophil concentration throughout the ACTH challenge in all animals, while no apparent changes were observed in monocytes, neutrophils, or lymphocytes. However, a study that observed the effects of cold stress and ACTH administration in Japanese Black steers saw that monocytes, eosinophils, and neutrophils increased, while lymphocytes decreased after ACTH was administered in a cold environment [35]. The differences between this study and the present study may be due to the additional cold stress that the Japanese Black steers were experiencing, or the age and breed differences in the animals utilized in the respective trials. Taken together, the results of this study demonstrate that some blood cell populations are affected slightly during a stressful period. However, intense stress or an increased cortisol response compared to the current study may affect blood cell populations more intensely. The neutrophil–lymphocyte ratio can be used as an indicator of stress in steers [36]. However, in the current study, stress did not impact N:L. Conversely, other studies have found that steers that experience stress have an increased N:L ratio [36,37,38]. The difference in results between the present study and other studies could be a result of actual cortisol concentration at harvest. In the present study, cortisol concentration at harvest was not different among harvest times.

The cortisol concentration relative to the ACTH challenge showed that the ACTH challenge did indeed induce a cortisol response, with peak concentrations observed between 0.5 and 1 h after ACTH was administered. Although the calves were the same breed, uniform in size, the same age, housed in the same environment, and sourced from one farm, differences in cortisol response were observed among harvest time groups 0.5 h after initiation of the ACTH challenge. However, cortisol concentration did not differ at harvest. This may be the result of differing behavior traits due to individual animal variation among the harvest time groups, as a difference in cortisol response was observed 0.5 h after the initiation of the ACTH challenge. The baseline cortisol concentration of the calves in the present study is similar to that in other studies [39,40,41]; however, the peak cortisol concentrations in the present study are lower compared to previous studies that performed an ACTH challenge on Swedish Red and White heifers and mature dairy cows, respectively [39,40]. Although the authors are unsure why lower peak cortisol concentrations were observed, it could be attributed to the environment the calves were housed in, the breed of the calves, or previous exposure to stressful events. Further, the dose of ACTH could have also been a factor.

Time of harvest and cortisol response were both shown to impact rectal temperature. Calves that had a low cortisol response had increased rectal temperatures. However, other studies have observed increased rectal temperatures during a stressful event. Stressful events like transportation and arrival at a new facility have been shown to increase rectal temperature [42]. Another study observed decreased rectal temperatures in calm Brahman bulls compared to temperamental- and intermediate-tempered bulls [43]. In addition, temperamental bulls had increased cortisol levels compared to calm bulls [43]. In Holstein heifers, rectal temperatures and cortisol concentrations were increased in heifers that were relocated via transportation compared to heifers that were not relocated [42]. It is important to note that although calves within the different harvest time groups displayed different rectal temperatures, the differences were not extreme and may not be of biological significance.

As molecular chaperones, SHSPs bind to and stabilize unstable proteins [7]. Various stressors result in the synthesis of HSPs [8]. The increase in cortisol prompts cellular adaptation, which is accomplished through the synthesis of HSPs [44]. The postmortem environment creates a “stressful” environment and in an attempt to maintain homeostasis, cells will increase the expression of SHSPs [33]. It has been shown that SHSPs are upregulated in different time frames based on the type of stress that the cell is undergoing, the intensity of the stress, and the specific SHSP [45]. Furthermore, during periods of stress, HSPβ1 can undergo post-translational modifications, which results in phosphorylation [7]. In response to oxidative stress, HSPβ1 can be phosphorylated, and in this form, it interacts with actin and protects it from fragmentation [46]. The SHSP HSPβ5 rapidly binds to and accumulates on myofibrils during stress to protect myofibrillar filament organization [47]. In the present study, we had initially hypothesized that calves that were given more time to recover following a stressful event would have a decreased expression of SHSPs. Relative HSPβ1 levels did not differ at harvest; however, they were increased after 14 d of aging in steaks from calves harvested 12 h after the ACTH challenge compared to other harvest times. Additionally, it is also important to note that no differences in HSPβ1 or P-HSPβ1 were found relative to cortisol response. Further, the expression of HSPβ5 did not differ based on the time of harvest nor cortisol response. Other studies have shown increased HSPβ1 abundance caused by various stressors. Heat stress caused by increased ambient temperatures in Brazil was measured using rectal temperature in Nelore and Caracu [48]. Increased heat stress resulted in increased relative HSPβ1 gene expression in the serum [48]. The expression of HSPβ1 mRNA in peripheral blood lymphocytes increased after 9 h of transportation, and gradually declined in Xia Nan cows [49]. To date, the authors are unaware of any research that has been completed on P-HSPβ1 or HSPβ5 in cattle. Additional research would be beneficial to determine how stress specifically impacts the abundance of HSPβ1 in skeletal muscle.

In the present study, MFI and troponin expression were used as an indication of tenderness development. The time of harvest affected MFI; however, cortisol levels 0.5 h after the challenge did not. The tenderness measurement, Warner–Bratzler shear force (WBSF), was not used in this study due to the inferior size of loins collected from the calves. A strong negative correlation between MFI and WBSF has been well documented [26,48]. In the present study, MFI was increased in calves that were harvested 24 h after the ACTH challenge, indicating that steaks from this group of calves may have an increased tenderness due to increased myofibrillar fragmentation. Other studies have observed that beef tenderness was affected following an acute stressor. Following 4 h of transportation, Hereford and Bradford steers were allowed to rest in lairage for various lengths of time, and steers that were in the long lairage group showed decreased WBSF values than steers from the short lairage group [49]. Another study showed that Nellore steers with increased chute scores or flightier temperaments had increased WBSF values [50]. Although MFI is shown to be correlated to WBSF and is a common measurement of tenderness, MFI is as a result of rather than a cause of postmortem aging [51]. As such, future studies need to include additional measurements, such as WBSF, to determine how the time of harvest following a stressful event affects end-product tenderness.

As mentioned above, troponin degradation was used in the present study to assess tenderness. The troponin complex is a principle regulatory component of the thin filament in skeletal muscle, and contains three subunits (troponin I, troponin C, and troponin T) [52]. The increased degradation of troponin is related to the increased tenderness of meat [53]. The present study used troponin I in the troponin degradation analysis. Troponin I is part of the tropomyosin–troponin complex and is an important component of myofibrils. In the present study, calves that were harvested 48 h after a stress exposure had an increased relative expression of troponin I in 14 d aged samples. In addition, calves that were harvested 24 h after stress exposure had a relatively large expression of troponin at harvest; however, this measurement was not significantly different from the other harvest groups. A study that characterized pre-harvest stress by dark, firm, and dry (DFD) meat in Rubi Gallega cattle observed that troponin C was increased in normal meat compared to the DFD meat in the *longissimus thoracis* (LT) [54]. In addition, researchers observed that DFD meat was more tender than normal meat [54]. Another study showed that Angus-influenced heifers designated for kosher slaughter are calmer than non-kosher steers; however, kosher heifers had increased WBSF values and an increased troponin T expression in the LL than non-kosher steers [55]. The effects of a stressful event prior to harvest on troponin expression and MFI have not been studied at length. More research in this area would be beneficial to fully elucidate this interaction.

The highly conserved protein, DJ1, is present in the cytoplasm as well as in the intracellular organelles and protects against oxidative stress [18]. In the present study, 0 calves that were allowed to rest for 24 h following a stressful event had the greatest expression of DJ1 at harvest and after 14 d of aging compared to calves in the other three harvest times. Little to no research has been conducted on the effects of premortem stress on DJ1 abundance in cattle. However, several studies have analyzed the relationship of DJ1 and the tenderness of meat. Some researchers have reported that tenderness was associated with the abundance of DJ1 [19,21]. Interestingly, the calves that were allowed to rest for 24 h after the ACTH challenge had the highest MFI compared to the other three treatment groups, which agrees with other studies that state that the protein abundance of DJ1 is linked to increased tenderness. In addition to DJ1 expression, serum TBARSs were used as a measurement of lipid peroxidation. Both the time of harvest and the cortisol response has a relationship with serum TBARSs concentration in the present study. Calves that were to be harvested 24 h after the ACTH challenge had an increased concentration of TBARSs in the serum 8 h after initiation of the challenge and at harvest. Researchers observed a three-fold increase in MDA in crossbred steers after transportation compared to before transportation [56]. In addition, another study observed a strong positive correlation between concentration of cortisol and TBARSs in calves after 2 h of transportation [57]. In cows infected with *Theileria annulata,* researchers observed increased lipid peroxidation measured in hemolysate compared to that of healthy cows [58]. It is apparent that certain stressors can increase the oxidation of tissues, which may affect meat quality [8,59]. However, more research would be beneficial to understand the effects of stress on the expression of DJ1 in skeletal muscle and the concentration of TBARSs in serum.

Steak color is an important quality that consumers consider when purchasing beef [13]. In the present study, time of harvest and cortisol response had an effect on a*, and time of harvest and cortisol response tended to affect b*. Cortisol response affected L*, such that calves that had a low cortisol response had increased L* values compared to calves with a high cortisol response. Calves that were harvested 24 h and 48 h after the ACTH challenge had steaks that were more red and yellow in color than steaks from calves that were harvested 12 h after the ACTH challenge. This is similar to what has been observed in previous studies. One study found that Hereford and Braford steers that were transported for 4 h had increased a* and b* values in the long lairage group; however, L* values were not significantly different between the short and long lairage times [49]. In another study, *Bos indicus* x *Bos taurus* cross cattle were allowed to rest in lairage for 3 h or 18 h after transportation, and steaks collected from the LL were aged for either 1 d or 14 d [60]. Researchers observed a lairage x aging interaction for a* and b* values, and cattle that were held in lairage for longer had increased a* and b* values in 14 d aged samples [60]. However, other researchers observed different results than the present study. A study utilizing Hungarian Simmental bulls observed increased L* values 24 h after harvest in bulls that were held in lairage for 48 h and 72 h compared to bulls that were held in lairage for 24 h; however, no differences were reported in a* values based on lairage time, and no differences in steak color 7 d after harvest were reported [61]. Friesian steers that were transported for 3 h or 16 h and kept in lairage for 3, 6, 12, or 24 h found that steers that were transported for 3 h and harvested 12 h after transportation had increased L* values compared to the other treatment groups [62]. These differences may be a result of differing lairage times and aging periods from the current study. The measurement of color closer to harvest would have been beneficial to better understand the observed color differences.

The measurement of pH is a good indicator of meat quality as it relates to the depletion of muscle glycogen [63,64,65]. Stressful events before harvest decrease glycogen reserves in the muscle, which, in turn, affects meat pH [63]. A normal pH for beef is approximately 5.5; however, dark-cutting beef may have a pH of 5.8 to 6.2 [66]. The pH of the LL was < 5.8 in the present study. In addition, the harvest time affected the pH of the LL. Calves that were harvested 48 h after the ACTH challenge produced meat that had greater pH values compared to the other harvest times. Interestingly, calves that were harvested 48 h after the ACTH challenge also had greater cortisol concentrations 0.5 h after the administration of ACTH, and greater a* values. Previous research has observed increased pH values in the *longissimus thoracis* of Friesian steers that were transported for 16 h compared to those transported for 3 h [62]. Additionally, another study observed the effects of using an electric prod before harvest on feedlot cattle and did not observe differences in the pH values of the meat produced from control animals compared to those that were handled with an electric prod [67]. Further, another study observed the effects of the temperament of steers on the pH and did not find any differences in pH values among the temperament groups [68]. The results of the present study may suggest that stressful events before harvest affect muscle pH; however, harvest time following a stressful event may have a greater impact on muscle pH.

## 5. Conclusions

The current study portrayed the complicated relationship between harvest time and cortisol response following a stressful event and how these factors may impact beef quality. From these data, we can observe that harvest time and the level of stress during a stressful event does impact the quality of beef in different ways. It can be generalized that animals that were harvested 12 h after the ACTH challenge resulted in an increased quality of beef due to the differences related to improved steak color, lower steak pH, decreased oxidation, and decreased MFI. The findings of this study are significant because they demonstrate that the time of harvest and cortisol response following a stressful event affects beef quality. Additional research, especially on industry-finished beef cattle, is needed to determine how premortem stress and cortisol response is involved in beef quality to provide consumers with beef of a consistent quality.

## Figures and Tables

**Table 1:** Description of primary bovine-specific antibodies and concentration used for Western blots.

Protein Name ^a^	Antibody Company	Host	Product Number	Primary Concentration	Secondary Concentration
HSPβ-1	Invitrogen ^b^	rabbit	PA1-25494	1:500	1:1000
PHSPβ-1	Invitrogen ^b^	rabbit	PA5-23340	1:2000	1:1000
HSPβ-5	Invitrogen ^b^	mouse	MAS-27708	1:4000	1:7500
DJ1	Abcam ^c^	rabbit	ab18257	1:1000	1:1000
Troponin	Invitrogen ^b^	rabbit	PA5-42108	1:500	1:1000

^a^ Heat shock protein β-1 (HSPβ-1), phosphorylated-HSPβ-1 (PHSPβ1), heat shock protein β-5 (HSPβ-5), protein deglycase (DJ1), troponin I (Troponin); ^b^ Invitrogen, Rockford, IL, USA; ^c^ Abcam, Cambridge, MA, USA.

**Table 2:** Relative abundance of proteins in the longissimus lumborum at either harvest or after 14 d of aging relative to the initial muscle biopsy collected prior to the adrenocorticotropic hormone (ACTH) challenge.

Harvest Time Relative to End of ACTH Challenge ^a^						
	2 h ^b^	12 h ^b^	24 h ^b^	48 h ^b^	SEM ^c^	*p*-Value ^x^
**Harvest**						
HSPβ1 ^d^	2.26	6.10	4.15	1.13	1.54	0.22
P-HSPβ1 ^e^	122.83 ^xy^	−86.06 ^x^	45.73 ^xy^	380.38 ^y^	107.03	**0.03**
HSPβ5 ^f^	−0.20	2.27	7.66	−0.41	3.75	0.67
DJ1 ^g^	6.44 ^xy^	−17.76 ^x^	73.25 ^y^	16.10 ^xy^	20.10	**0.002**
Troponin ^h^	−24.52	127.26	995.10	162.52	320.74	0.22
**14 d Aged**						
HSPβ1 ^d^	−0.02	5.48	2.05	1.94	1.10	**0.03**
P-HSPβ1 ^e^	0.26 ^x^	0.97 ^y^	0.54 ^xy^	0.16 ^x^	0.15	**0.001**
HSPβ5 ^f^	−0.73	11.05	25.66	−2.31	9.82	0.43
DJ-1 ^g^	2.55 ^x^	−5.18 ^x^	54.57 ^y^	12.32 ^x^	7.72	**<0.0001**
Troponin— Entire Band ^i^	1.07 ^xy^	0.19 ^x^	2.98 ^xy^	3.37 ^y^	0.73	**0.01**
Troponin— Lower Band ^j^	0.31 ^x^	0.21 ^x^	0.48 ^xy^	1.34 ^y^	0.24	**0.02**

^a^ Treatment groups consisted of four groups of castrated Holstein calves (103.5 ± 1.6 kg) that were harvested at different time points (2 h, 12 h, 24 h, and 48 h; *n* = 10) following the ACTH challenge given at a dose of 0.1 IU/kg of body weight. ^b^ Columns are least square means for the relative abundance of each protein. Protein abundance was calculated by dividing the initial protein abundance of the skeletal muscle biopsy by the abundance in the initial skeletal muscle biopsy. This calculation was carried out for samples collected both at harvest and after 14 days of aging. ^c^ SEM = standard error of the mean. ^d^ Heat shock protein β1. ^e^ Phosphorylated heat shock protein β1. ^f^ Heat shock protein β5. ^g^ Protein deglycase. ^h^ Troponin at harvest is one large band that is intact. ^i^ Entire band of troponin is measured by analyzing protein abundance of all troponin bands that appear after degradation following harvest. ^j^ Lower band of troponin indicates protein abundance of the lower of two bands of protein, which indicates degradation. ^x,y^ Values within a row with different letters (i.e. x, y, etc.) are different (*p* < 0.05) from one another.

**Table 3:** Relative abundance of proteins in the longissimus lumborum at either harvest or after 14 d of aging relative to the initial muscle biopsy collected prior to the adrenocorticotropic hormone (ACTH) challenge between steers differing in cortisol response.

Cortisol Response ^a^				
	Low ^b^	High ^b^	SEM ^c^	*p*-Value ^x^
**Harvest**				
HSPβ1 ^d^	2.29	4.14	1.15	0.29
P-HSPβ1 ^e^	210.32	21.12	80.04	0.15
HSPβ5 ^f^	0.52	4.14	2.80	0.40
DJ1 ^g^	38.77	0.24	15.03	0.07
Troponin ^h^	188.44	441.74	239.86	0.49
**14 d Aged**				
HSPβ1 ^d^	1.52	3.20	1.67	0.19
P-HSPβ1 ^e^	0.45	0.51	0.11	0.81
HSPβ5 ^f^	2.14	14.69	7.34	0.25
DJ-1 ^g^	23.49	8.64	5.99	0.08
Troponin— Entire Band ^i^	2.14	1.66	0.54	0.52
Troponin— Lower Band ^j^	0.56	0.61	0.18	0.88

^a^ Treatment groups consisted of four groups of castrated Holstein calves (103.5 ± 1.6 kg) that were harvested at different time points (2 h, 12 h, 24 h, and 48 h; *n* = 10) following the ACTH challenge given at a dose of 0.1 IU/kg of body weight. ^b^ Columns are least square means for the relative abundance of each protein. Protein abundance was calculated by dividing the initial protein abundance of the skeletal muscle biopsy by the abundance in the initial skeletal muscle biopsy. This calculation was carried out for samples collected both at harvest and after 14 days of aging. ^c^ SEM = standard error of the mean. ^d^ Heat shock protein β1. ^e^ Phosphorylated heat shock protein β1. ^f^ Heat shock protein β5. ^g^ Protein deglycase. ^h^ Troponin at harvest is one large band that is intact. ^i^ Entire band of troponin is measured by analyzing protein abundance of all troponin bands that appear after degradation following harvest. ^j^ Lower band of troponin indicates protein abundance of the lower of two bands of protein, which indicates degradation. ^x^ Values within a row with different letters are different (*p* < 0.05) from one another.

## Data Availability

The data presented in this manuscript are available following a request to the corresponding author.
